# Enhancing LiDAR Mapping with YOLO-Based Potential Dynamic Object Removal in Autonomous Driving

**DOI:** 10.3390/s24237578

**Published:** 2024-11-27

**Authors:** Seonghark Jeong, Heeseok Shin, Myeong-Jun Kim, Dongwan Kang, Seangwock Lee, Sangki Oh

**Affiliations:** 1 Propulsion Division, GM Korea Company, Incheon 21344, Republic of Korea; seonghark.jeong@gm.com; 2 Convergence Major for Intelligent Drone, Sejong University, Seoul 05006, Republic of Korea; hsshin9017@sejong.asssc.kr; 3 Graduate School of Automotive Mobility, Kookmin University, Seoul 02707, Republic of Korea; kmjcr7@kookmin.ac.kr (M.-J.K.); energy@kookmin.ac.kr (S.L.); 4 Hanwha Aerospace, Seongnam 13488, Republic of Korea; dongwan.kang@hanwha.com; 5 Department of Automotive Engineering, Gyeonggi University of Science and Technology, Siheung 15073, Republic of Korea

**Keywords:** LiDAR, NDT, autonomous vehicle, semantic segmentation, localization, map matching, DeepLabV3+, YOLOv4, sensor fusion

## Abstract

In this study, we propose an enhanced LiDAR-based mapping and localization system that utilizes a camera-based YOLO (You Only Look Once) algorithm to detect and remove dynamic objects, such as vehicles, from the mapping process. GPS, while commonly used for localization, often fails in urban environments due to signal blockages. To address this limitation, our system integrates YOLOv4 with LiDAR, enabling the removal of dynamic objects to improve map accuracy and localization in high-traffic areas. Existing methods using LiDAR segmentation for map matching often suffer from missed detections and false positives, degrading performance. Our approach leverages YOLOv4’s robust object detection capabilities to eliminate potentially dynamic objects while retaining static environmental features, such as buildings, to enhance map accuracy and reliability. The proposed system was validated using a mid-size SUV equipped with LiDAR and camera sensors. The experimental results demonstrate significant improvements in map-matching and localization performance, particularly in urban environments. The system achieved RMSE (Root Mean Square Error) reductions compared to conventional methods, with RMSE values decreasing from 0.9870 to 0.9724 in open areas and from 1.3874 to 1.1217 in urban areas. These findings highlight the ability of the Vision + LiDAR + NDT method to enhance localization performance in both simple and complex environments. By addressing the challenges of dynamic obstacles, the proposed system effectively improves the accuracy and robustness of autonomous navigation in high-traffic settings without relying on GPS.

## 1. Introduction

Autonomous vehicle localization has traditionally depended on GPS for positioning. However, GPS-based localization, even with dual or RTK-GPS setups, can fail in urban environments due to tall buildings obstructing signals, making it unreliable for high-precision tasks. GPS errors in such environments can be severe, making it difficult to rely solely on GPS for accurate localization. As a result, modern autonomous systems require the use of additional sensors, such as cameras and LiDAR, to achieve accurate localization [1].

LiDAR-based localization has gained significant attention due to its ability to accurately measure distances using the time of flight of laser beams. Two popular approaches for LiDAR-based map matching are the Normal Distributions Transform (NDT) and the Iterative Closest Point (ICP) algorithms. ICP provides highly accurate point-to-point matching but is computationally expensive and prone to errors in complex environments, particularly when points do not correspond correctly. NDT, which works by transforming point clouds into normal distributions, is more computationally efficient and better suited for localization in dynamic environments. Consequently, we use the NDT algorithm in this study for its robustness and efficiency in complex settings [2].

Despite these advantages, using NDT in real-world scenarios presents challenges. Current methods using LiDAR segmentation for vehicle removal suffer from inaccuracies, leading to missed detections and false positives that degrade map-matching performance. This limitation is particularly problematic in high-traffic areas, where segmentation errors can lead to inaccuracies in localization and overall system performance.

To mitigate these issues, we propose a novel approach that combines LiDAR with a camera-based YOLO algorithm to detect and remove dynamic objects, specifically vehicles, from the mapping process. The YOLOv4 algorithm, known for its real-time object detection capabilities, accurately identifies moving vehicles, which are then excluded from the map. By removing these dynamic objects, we generate a more accurate and reliable map that better represents the static environment, enhancing the localization performance without the need for GPS sensors. This method is particularly effective in high-density urban environments, where moving vehicles can significantly degrade the localization accuracy if not properly handled [3].

In this paper, we validate the proposed method using a mid-size SUV equipped with both LiDAR and camera sensors. Our experimental results demonstrate significant improvements in mapping accuracy and localization performance, especially in high-traffic environments. By leveraging the camera-based YOLO algorithm, we effectively address the limitations of traditional LiDAR-based mapping methods, paving the way for more reliable and efficient autonomous vehicle localization in complex urban settings.

## 2. Methodology

### 2.1. ICP Algorithm

The Iterative Closest Point (ICP) algorithm operates by iteratively searching for pairs of nearby points between two scans and minimizing the sum of distances between all point pairs. However, ICP has two primary limitations. First, it is point-based, meaning that it does not take advantage of the local surface geometry around each point. Second, in the main loop, the nearest-neighbor search is computationally expensive. To improve search speed, efficient data structures such as k-d trees, which support approximate nearest-neighbor searches, can be used [4]. Despite this, the search process remains the main bottleneck in the algorithm’s runtime [5].

If the point pairs found in the first step of the algorithm truly correspond to the same points on the scanned surfaces, the computed transformation will be accurate. However, because the nearest point is used as a guess for correspondence, it is crucial to detect and filter out incorrect correspondences, keeping only the most reliable ones. One strategy for handling this is to apply a form of “soft” outlier rejection by assigning different weights to point pairs [6]. In this approach, more weight is given to point pairs that are more likely to contribute to the final result, while less weight is assigned to those more likely to be incorrect correspondences. A common criterion for weighting is the relative distance between points. The weight ω of a correspondence between points x and y can be adjusted in proportion to their distance, with pairs that have larger distances being down-weighted.

(1) $$\omega =1-\frac{\left|x-y\right|}{max\left|x_{i}-y_{i}\right|}$$

However, we found that linear weighting based on distance did not improve the results for the data. For example, in datasets such as tunnels or corridors, distance-based weighting can degrade the performance. Most of the points along walls and ceilings are typically well-aligned, meaning that their influence can overwhelm the impact of point pairs with larger distances, which may correspond to important features like edges or corners. For this reason, we chose to assign equal weights to all point pairs [7].

### 2.2. NDT Algorithm

The Normal Distributions Transform (NDT) is a statistical method used for aligning two point clouds, commonly referred to as the Target and Source point clouds. The Target point cloud serves as the reference, while the Source point cloud is transformed through rotation and translation to match the Target. Unlike the Iterative Closest Point (ICP) algorithm, which matches individual points between the point clouds, NDT takes a statistical approach by modeling local regions of the point cloud as probability distributions. This method is widely used in applications such as LiDAR-based localization and mapping, where precise and efficient alignment of 3D data is required [8].

In this paper, we use NDT for generating a point cloud map, which is then utilized for LiDAR-based localization. NDT works by dividing the 3D space into grids, computing statistical characteristics (mean and variance) for each grid, and then matching the transformed point cloud to the reference cloud using these characteristics.

#### 2.2.1. NDT Algorithm Operation

Given a 3D point cloud $M=\{m_{1},\;m_{2},\ldots ,m_{k}\}$, the 3D space is first subdivided into a grid of a predefined size. Each point in the point cloud is then assigned to its corresponding grid cell. For each grid cell, the mean value μ and variance P of the registered points are computed and stored. The corresponding formulations are described below.

- Mean Value (μ)

For a grid cell G, let the set of points within the cell be $m_{1},\;m_{2},\ldots ,m_{k}$, where each point is represented by its 3D coordinates $m_{i}=(x_{i}{,y}_{i},z_{i})$. The mean value $\mu_{G}$ of the points in the grid cell G is computed as follows:

(2) $$\mu_{G}=\left(\frac{1}{k}\sum_{i=1}^{k}x_{i},\;\frac{1}{k}\sum_{i=1}^{k}y_{i},\frac{1}{k}\sum_{i=1}^{k}z_{i}\right)$$

where *k* represents the number of points in the grid cell.

- Variance (P)

The variance $P_{G}$ of the points in the grid cell G is calculated for each coordinate axis as follows:

(3) $$P_{G}=\left(\frac{1}{k}\sum_{i=1}^{k}{\left(x_{i}-\bar{x}\right)}^{2},\;\frac{1}{k}\sum_{i=1}^{k}{\left(y_{i}-\bar{y}\right)}^{2},\frac{1}{k}\sum_{i=1}^{k}{\left(z_{i}-\bar{z}\right)}^{2}\;\right)$$

where $\bar{x},\;\bar{y},\;\bar{z}$ represent the mean values of the x, y, and z coordinates, respectively, computed using the formula for the mean.

#### 2.2.2. Scan Matching Using NDT

The goal of scan matching using NDT is to find the pose information of the source point cloud that allows it to be accurately aligned with the target point cloud. Let the input source point cloud be represented as $X=\{x_{1},\ldots ,\;x_{n}\}$. The likelihood function is expressed as shown in Equation (4):

(4) $$\hat{p}\left(x\right)=\sum_{k=1}^{n}p(T\left(\hat{p},{\hat{x}}_{k}\right))$$

This represents the likelihood of a transformed point $\hat{x}$. The aim is to find the optimal value for the source point cloud’s pose. However, the normal distribution used here can be highly sensitive to outliers. To robustly address this, the negative log-likelihood of Equation (4) is taken, resulting in Equation (5):

(5) $$\hat{p}\left({\hat{x}}_{k}\right)=-d_{1}exp\left(-\frac{1}{2d_{2}}{\left({\hat{x}}_{k}-\mu_{k}\right)}^{T}\sum_{k}^{-1}\left({\hat{x}}_{k}-\mu_{k}\right)\right)$$

Here, the constants $d_{1}$ and $d_{2}$ can be derived using the method proposed in [9]. Using this approach, the final score function of NDT, $s(\hat{p})$, can be expressed as follows:

(6) $$s(\hat{p})=-\sum_{k=1}^{n}p(T\left(\hat{p},{\hat{x}}_{k}\right))\;$$

NDT aims to minimize the score function in Equation (6) to optimize the function, and the parameter $\hat{p}$ is determined using Newton’s method, as described in Equation (7).

(7) $$H∆\hat{p}=-\hat{g}$$

In Equation (7), H represents the Hessian matrix of $s(\hat{p})$, and $\hat{g}$ is the gradient vector. The value $∆\hat{p}$ is iteratively added to the current pose estimate in each iteration, as shown in Equation (8):

(8) $$\hat{p}\leftarrow \hat{p}+∆\hat{p}$$

Furthermore, assuming that:

$${\hat{x}\prime }_{k}=T\left(\hat{p},{\hat{x}}_{k}\right)-\mu_{k}$$

The Hessian matrix and gradient vector can be computed using Equations (9) and (10).

(9) $$g_{i}=\frac{\partial_{p_{i}}}{\partial_{s}}\sum_{k=1}^{n}d_{1}d_{2}{\hat{x}\prime }_{k}^{T}\sum_{k}^{-1}\frac{\partial_{p_{i}}}{\partial_{{\hat{x}\prime }_{k}}}exp\left(-\frac{1}{2d_{2}}{\hat{x}\prime }_{k}^{T}\sum_{k}^{-1}{\hat{x}\prime }_{k}\right)$$

(10) $$H_{ij}=\frac{{\partial^{2}}_{s}}{\partial_{p_{i}}\partial_{p_{j}}}\sum_{k=1}^{n}d_{1}d_{2}exp\left(-\frac{1}{2d_{2}}{\hat{x}\prime }_{k}^{T}\sum_{k}^{-1}{\hat{x}\prime }_{k}\right)\left(-d_{2}{\hat{x}\prime }_{k}^{T}\sum_{k}^{-1}\frac{\partial p_{i}}{\partial {\hat{x}\prime }_{k}}\right)\;\left(\frac{\partial p_{j}}{\partial {\hat{x}\prime }_{k}}\right)+\cdots$$

### 2.3. Sementic Segmentation (DeepLabV3+)

**Figure 1:**
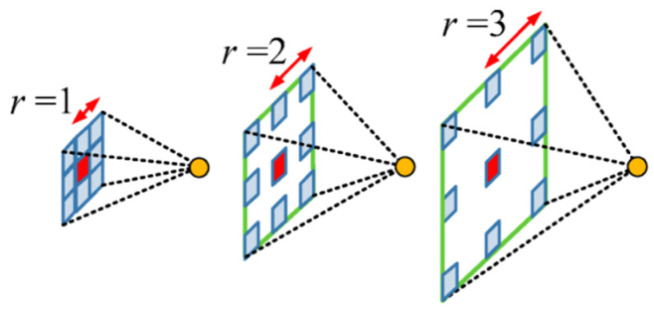
Illustration of the atrous convolution mechanism. Different colors (blue, green, and red) represent receptive fields for dilation rates of 1, 2, and 3, respectively.

To effectively detect dynamic objects, such as vehicles, from LiDAR-based mapping, we utilize DeepLabV3+, an advanced semantic segmentation model. DeepLabV3+ leverages atrous convolution (also known as dilated convolution) to preserve high-resolution spatial features while expanding the receptive field. The atrous convolution mechanism used to maintain spatial resolution is illustrated in Figure 1 [10].

This allows for more precise object segmentation without increasing the computational complexity, which is critical for real-time applications in environments with complex object distributions. Additionally, depth-wise separable convolution is employed to reduce computational costs while maintaining performance levels, thus improving efficiency without sacrificing segmentation accuracy [11].

**Figure 2:**
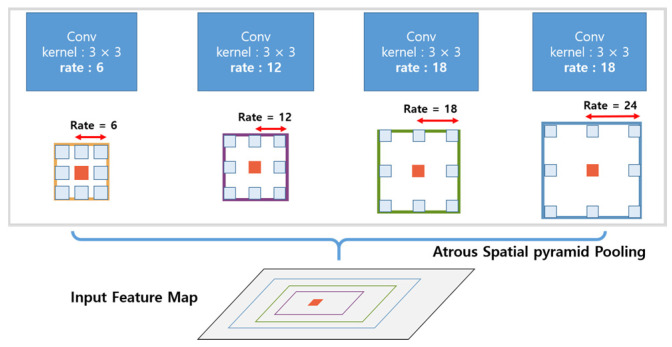
Diagram of the Atrous Spatial Pyramid Pooling (ASPP) module for multi-scale feature extraction. Different colors (blue, orange, green, and purple) indicate the receptive fields for dilation rates of 6, 12, 18, and 24, respectively.

DeepLabV3+ improves segmentation by incorporating an Atrous Spatial Pyramid Pooling (ASPP) module, which aggregates multi-scale context information efficiently. The multi-scale feature extraction capabilities of the ASPP module are shown in Figure 2.

This feature makes it especially suitable for differentiating dynamic obstacles from the static environment in high-density urban areas. By using ASPP, the model captures features at multiple scales, enabling it to perform well in scenarios with objects of varying sizes and distances.

The encoder–decoder architecture of DeepLabV3+ efficiently reconstructs fine object boundaries for improved segmentation.

**Figure 3:**
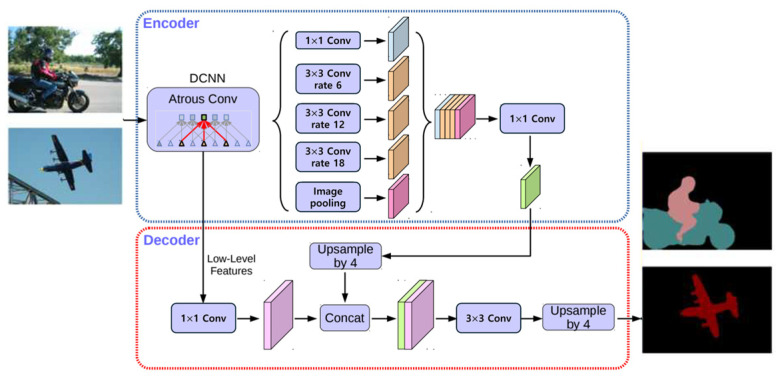
Flowchart of DeepLabV3+ architecture highlighting the encoder–decoder structure and Xception backbone.

While the decoder works to predict object boundaries more accurately, the encoder utilizes a modified Xception backbone that has been optimized for semantic segmentation tasks. This architecture shares similarities with the U-Net structure but offers improved efficiency and accuracy, particularly when dealing with complex object segmentation in real-world environments. The full architecture of DeepLabV3+, highlighting its encoder–decoder structure and Xception backbone, is detailed in Figure 3.

By integrating DeepLabV3+ into our system, we ensure that moving vehicles are accurately segmented and removed from the LiDAR map [12,13].

### 2.4. YOLOv4 Algorithm

DeepLabV3+ is widely recognized for its powerful semantic segmentation capabilities. However, despite its advanced design, there are instances where vehicles may not be detected accurately, particularly in dynamic environments with high traffic. Such missed detections can cause significant errors during map matching, leading to inaccuracies in the mapping process. To address this issue, we incorporated YOLOv4 algorithm into the map generation process. By leveraging the strengths of YOLOv4, we aim to ensure that undetected vehicles are removed effectively, thereby enhancing the accuracy of map matching. YOLOv4, which excels in the detection of vehicles and pedestrians, was specifically employed in this study to improve vehicle detection and removal, focusing on enhancing the overall performance of map matching in complex, high-traffic scenarios.

In contrast to multi-stage detection models, YOLOv4 performs object classification and bounding box regression simultaneously in a single pass through the network, making it much faster than traditional approaches like Faster R-CNN, which separates the region proposal and classification stages [14].

The following content presents Table 1 and the explanation for the reasons behind using YOLOv4 [15].

- Stability and Compatibility

YOLOv4 ensures real-time processing stability through the use of Cross-Stage Partial Networks (CSP) and a Path Aggregation Network (PAN). While YOLOv5 and YOLOv7 deliver strong performance, they require significantly higher computational resources (FLOPs) in specific scenarios such as urban environments with high-density traffic. This makes them less efficient for systems with resource constraints.

- Optimization of Computational Resources

YOLOv4 maintains high stability while delivering a real-time analysis at 62 FPS with relatively lower computational requirements. By contrast, YOLOv5 and YOLOv7 achieve peak performance only in high-end GPU environments, making YOLOv4 the more suitable choice in terms of efficiency.

- Specialization for Urban Environments

YOLOv4 excels in detecting vehicles across varying object scales, making it particularly effective for map-matching and dynamic object removal tasks. Although YOLOv7 achieves higher mAP and performs well in general object detection tasks, YOLOv4 is better suited for specific applications like static and dynamic vehicle removal in urban environments.

**Figure 4:**
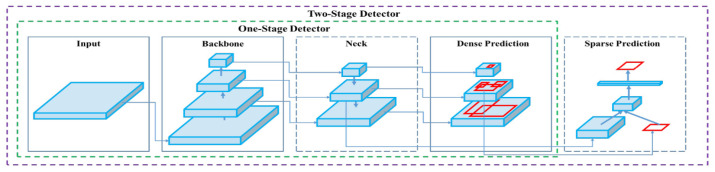
YOLOv4 detection pipeline illustrating object classification and localization. (Red boxes indicate the predicted bounding boxes for object localization).

The YOLOv4 network architecture is inspired by Google’s GoogLeNet, using a convolutional neural network (CNN) structure consisting of 24 convolutional layers followed by two fully connected layers. The convolutional layers are responsible for feature extraction, while the fully connected layers predict object classes and bounding box coordinates. Unlike GoogLeNet’s Inception modules, YOLOv4 employs a combination of 1 × 1 reduction layers and 3 × 3 convolutional layers for efficient feature extraction. The Fast YOLOv4 variant reduces the architecture to nine convolutional layers, further optimizing the network for real-time detection without sacrificing accuracy. Figure 4 illustrates the core architecture of the YOLOv4 detection pipeline, showing the convolutional layers and the prediction tensor output.

YOLOv4, with its CSP-DarkNet53 backbone and Path Aggregation Network (PAN), enhances the object detection performance by improving both the accuracy and computational efficiency, making it particularly suitable for dynamic environments. The model’s architecture effectively removes redundant information during training through CSP connections, which contributes to its high speed and accuracy, which is essential for the real-time detection and removal of vehicles to improve map-matching accuracy [16].

**Figure 5:**
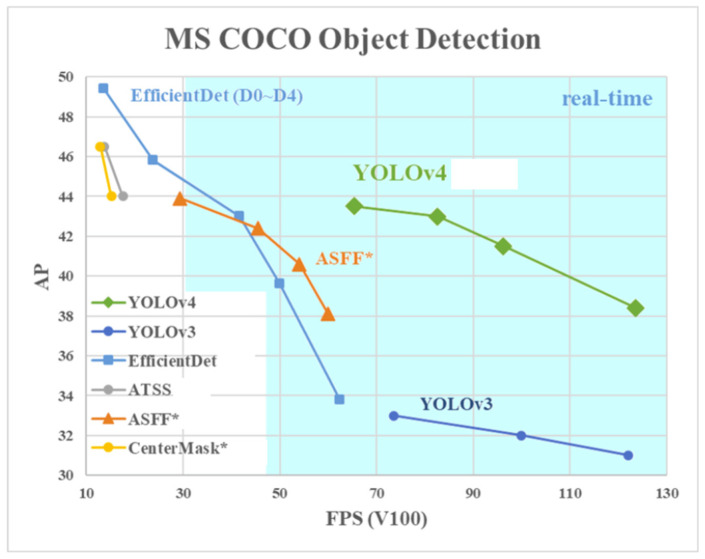
Benchmark comparison of YOLOv4 with other detection algorithms highlighting speed and accuracy improvements.

Figure 5 illustrates the benchmark data from the YOLOv4 paper, showcasing the relationship between FPS (frames per second) and AP (average precision) for several state-of-the-art object detection algorithms. The comparison algorithms, including RetinaMask, Cascade RCNN, and TridentNet, were selected based on their recognition as leading methods during YOLOv4’s development. These models were evaluated for their ability to balance accuracy (AP) and real-time inference capabilities (FPS), which are critical metrics for applications requiring real-time processing in dynamic environments. The results in Figure 5 highlight YOLOv4’s superior balance of speed and accuracy compared to these algorithms, further validating its selection for this study.

The YOLOv4 model used in this study was pre-trained on the ImageNet dataset and fine-tuned for vehicle detection. To optimize its performance, the final layer employs a linear activation function, while earlier layers utilize leaky ReLU to enhance gradient propagation. The model minimizes sum-squared error (SSE) loss during training, which, while simplifying the optimization process, does not differentiate between classification and localization errors. To address this, an IoU-based bounding-box selection method was incorporated, prioritizing bounding boxes with higher Intersection over Union (IoU) scores to improve detection accuracy [17].

### 2.5. LiDAR and Camera Calibration with Dynamic Object Removal

#### 2.5.1. Camera and LiDAR Calibration

Accurate sensor fusion relies heavily on the precise calibration of individual sensors. Particularly, the camera introduces inherent distortions due to its structural characteristics, such as the image sensor, lens, and focal length, as it projects three-dimensional (3D) spatial data onto two-dimensional (2D) images. To correct these distortions, camera calibration is required, which involves determining both intrinsic and extrinsic parameters. These parameters enable the transformation of 3D spatial coordinates into the camera’s coordinate system, which is a crucial process for accurate sensor fusion [18,19].

- Camera Calibration

Camera calibration consists of estimating the intrinsic parameters, including focal length, principal point, and skew coefficient, which are determined by the camera’s internal physical properties. These intrinsic parameters are major contributors to image distortion. Additionally, extrinsic parameters, which describe the camera’s position and orientation in 3D space, are crucial for defining the spatial relationship between the camera coordinate system and the world coordinate system. Specifically, the extrinsic parameters consist of the camera’s position and rotation, which allow the transformation of 3D points in space into 2D coordinates on the camera image. This calibration process lays the foundation for sensor fusion by accurately aligning the camera’s output with other sensor data.

- LiDAR Calibration

After correcting for distortions through the camera’s intrinsic calibration, calibration between the camera and LiDAR sensor is necessary to ensure accurate alignment between the two systems. This step involves determining the geometric relationship between the sensors by calculating the rotation matrix and translation vector. Typically, a chessboard pattern is used, with its position varied to compute the relative orientation and position of the two sensors. The resulting rotation matrix and translation vector facilitate the transformation of 3D point cloud data from LiDAR into the camera’s 2D image plane. This transformation enables the assignment of LiDAR-derived depth information to each pixel in the camera image, which is vital for object detection and tracking applications.

- Sensor Fusion

Once the calibration between the camera and LiDAR is complete, sensor fusion can be further enhanced by applying the YOLOv4 algorithm. This algorithm detects objects within the camera image, providing both class and positional information. The detected objects in the camera’s field of view are then combined with distance information obtained from LiDAR, enabling the precise calculation of the object’s 3D position and distance from the sensor system. The process of projecting 3D LiDAR points (X,Y,Z) onto 2D pixel coordinates (x,y) can be described using a calibration matrix A, a 3 × 3 rotation matrix R, and a 3 × 1 translation vector *t*. This transformation is expressed in Equation (11), while the final calibration formula is presented in Equation (12).

(11) $$X=A\left[R\left|t\right|\right]X_{w}$$

(12) $$\left[\begin{array}{c} x\\ y\\ 1 \end{array}\right]=\left[\begin{array}{ccc} \alpha_{x} & s & x_{0}\\ 0 & \alpha_{y} & y_{0}\\ 0 & 0 & 1 \end{array}\right]\left[\begin{array}{cccc} r_{11} & r_{12} & r_{13} & t_{1}\\ r_{21} & r_{22} & r_{23} & t_{2}\\ r_{31} & r_{32} & r_{33} & t_{3} \end{array}\right]\left[\begin{array}{c} x_{W}\\ y_{W}\\ z_{W}\\ 1 \end{array}\right]$$

#### 2.5.2. LiDAR Segmentation for Dynamic Object Removal

In order to accurately identify and classify objects within raw LiDAR data, the DeepLabV3+ network was modified to specifically optimize it for LiDAR input. This modification was necessary due to the inherent differences between standard image data, for which DeepLabV3+ was originally designed, and the 3D point cloud data generated by LiDAR sensors. By adapting the network to process LiDAR data more effectively, the proposed model is able to perform semantic segmentation tasks in a manner that aligns with the characteristics of 3D point clouds.

The modified DeepLabV3+ network was trained and validated using a substantial dataset comprising 43,000 pieces of Semantic KITTI data, which includes high-quality, annotated LiDAR point cloud scans [20]. The training process aimed to fine-tune the network’s ability to distinguish between various object classes within a 3D environment, particularly focusing on vehicular objects. Validation tests were conducted to assess the generalizability and robustness of the model across diverse scenarios involving dynamic and static objects. The results of these performance evaluations are summarized in Table 2. For these experiments, 16 distinct object classes were employed, representing various types of vehicles, pedestrians, and environmental features [21,22,23].

To evaluate the effectiveness of the proposed semantic segmentation approach, Intersection over Union (IoU) was utilized as the primary performance metric. IoU is a widely accepted measure in segmentation tasks, as it quantifies the accuracy of object detection by comparing the overlap between the predicted segmentation and the ground truth. Specifically, IoU is defined as the ratio of the intersection area to the union area of the predicted and actual segments. A higher IoU indicates a closer match between the predicted segmentation and the actual object boundary.

The DeepLabV3+ method achieved a mean IoU of 65.72, which is indicative of excellent performance relative to other state-of-the-art models in similar tasks. This result underscores the network’s capacity to accurately segment and classify objects in the complex, unstructured environments typicsssally encountered in autonomous vehicle applications. For reference, an IoU value of 100 corresponds to a perfect match between the predicted and actual segmentation, whereas an IoU of 0 indicates no overlap. The high mean IoU achieved by the modified DeepLabV3+ demonstrates its effectiveness in processing LiDAR data for vehicle detection and its potential applicability in real-world scenarios where robust object detection is critical for autonomous driving systems.

#### 2.5.3. Vision Object Detection for Dynamic Objssect Removal

To evaluate the classification accuracy of the object detection component, we conducted benchmark tests using the MS COCO dataset, as shown in Table 3. The object detection tasks were performed on the Nvidia Jetson AGX Xavier board, and we optimized the network using TensorRT for improved efficiency. Our benchmark tests revealed a trade-off between input size, detection accuracy, and processing speed. To balance human-like perceptual speed with reasonable accuracy, we set the image input size to 512 × 512 for our experiments [24].

To further evaluate the performance of the detector, we randomly selected 300 frames containing vehicles and assessed the detection accuracy of the targeted objects. The test environment’s ground truth included 1341 vehicles comprising cars, buses, and trucks. Detection was considered correct when the IoU (Intersection over Union) was 0.5 or greater. The resulting accuracy is summarized in Table 4. As the detector is integrated with a tracking algorithm, the overall detection performance can be expected to be even more robust than indicated by these metrics.

### 2.6. Proposed Method for Improving Map-Matching Performance

As discussed in the previous sections, DeepLabV3+ has demonstrated superior performance when applied to LiDAR data for object detection compared to other existing algorithms. In this study, the vehicle data detected by DeepLabV3+ is utilized to improve the performance of the map-matching process. However, relying solely on LiDAR data for map matching introduces certain limitations. For example, LiDAR can mistakenly classify non-vehicle objects, such as container boxes, as buses, leading to the erroneous removal of valid map data [25].

To address this issue, we propose a novel map-matching approach that combines both LiDAR and camera data to improve accuracy. By leveraging the complementary strengths of these two sensors, the proposed method mitigates the risk of false detections and enhances the map-matching performance. Specifically, the camera is used to assist in vehicle detection, employing the YOLOv4 algorithm to filter out non-vehicular objects. In this work, we apply YOLOv4, as described in Section 2.5.3, to achieve reliable object detection for the purpose of map matching [26].

The proposed method can be described as follows:

- Threshold Percentage Calculation:

To classify objects detected by both LiDAR and the camera, we calculate a threshold percentage based on the individual accuracies of both systems. This threshold, x, is computed as follows:

(13) $$x=\frac{L_{a}\times C_{a}}{100\times 100}$$

where $L_{a}$ and $C_{a}$ are the respective accuracies of the LiDAR and camera systems.

Next, we introduce a gain factor to balance the contribution of both sensors calculated as follows:

(14) $$gain=\frac{L_{a}}{C_{a}}$$

Using the calculated threshold and gain, an object is classified as a vehicle if:

(15) $$(C_{a}\times gain)\times L_{a}>x$$

In this case, the object is removed from the map. Conversely, if the condition is not met:

(16) $$(C_{a}\times gain)\times L_{a}<x$$

the object is classified as a false positive and is retained in the map.

## 3. Experiment and Results

### 3.1. Experimental Environment

**Figure 6:**
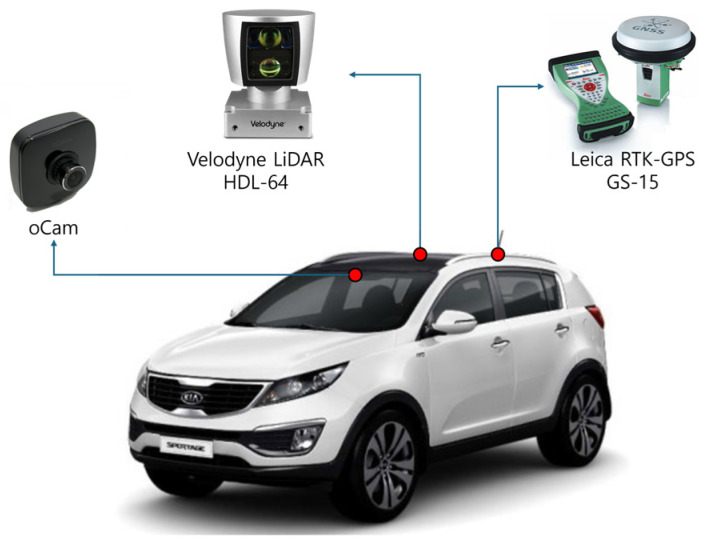
Experimental setup featuring a mid-sized SUV equipped with Ouster LiDAR, O-cam, and Leica GS15 GPS for map-matching validation.

For the testing and validation of the proposed map-matching algorithm, we utilized the mid-sized SUV depicted in Figure 6. The experiment involved removing vehicle data using a 64-channel Ouster LiDAR and an O-cam mounted on the SUV. Additionally, localization was performed using a Leica GS15 GPS, which was also installed on the vehicle. This setup ensured accurate data collection and enabled effective vehicle removal for testing the map-matching algorithm under real-world conditions.

The National Assembly and 63 Building areas were chosen as experimental locations due to their distinct environments. The National Assembly area, categorized as an open environment, features wide streets, low vehicle density (approximately 30 vehicles per square kilometer during testing), and minimal obstructions, making it ideal for evaluating the system’s map-matching performance in less-complex scenarios with sparse landmarks. By contrast, the 63 Building area, categorized as an urban environment, is characterized by high vehicle density (approximately 120 vehicles per square kilometer during peak hours), narrow street layouts, and dense high-rise buildings, creating a GPS-denied zone with frequent dynamic obstacles.

**Figure 7:**
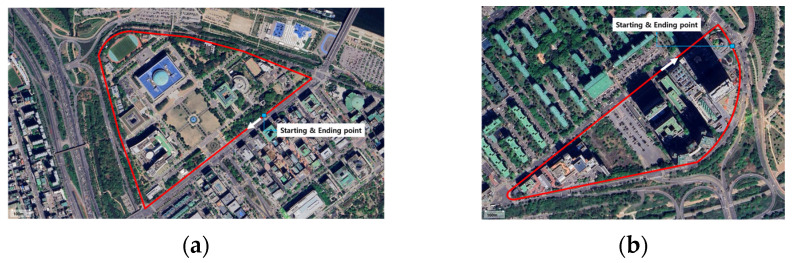
Experimental locations: (**a**) National Assembly and (**b**) 63 Building maps.

As shown in Figure 7, These contrasting environments allowed us to comprehensively evaluate the proposed system’s performance under diverse conditions: one focusing on precision in sparse-feature settings and the other on robustness in highly dynamic and obstructed urban scenarios. The system consistently demonstrated improved map-matching performance in both environments, with slight variations in RMSE due to environmental complexity. This analysis highlights the relevance of these test sites to the study’s objectives. 

**Figure 8:**
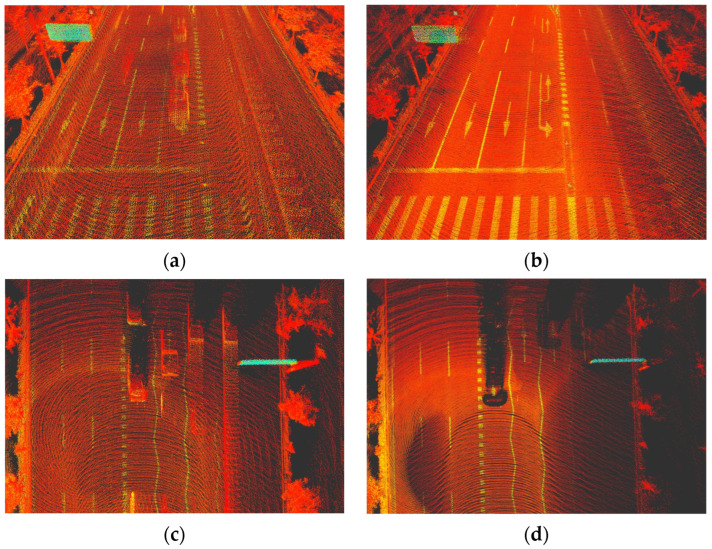
Before-and-after LiDAR maps: (**a**,**c**) raw LiDAR maps with vehicles; (**b**,**d**) processed maps after dynamic object (vehicle) removal using DeepLabV3+.

By applying this combined approach, the system reduces the likelihood of false positives, such as the incorrect removal of static objects that are mistakenly identified as vehicles by LiDAR. The before-and-after comparison of LiDAR maps, showing the impact of dynamic object removal, is shown in Figure 8. The use of both sensors in tandem allows for a more robust and accurate map-matching process, ensuring that map data remain reliable and accurate even in complex environments.

### 3.2. Result of Map Matching

The experiments were conducted in two distinct environments: an Open Area near the National Assembly with wide-open surroundings and an Urban Area dense with high-rise buildings. The Open Area is characterized by a 2.5 km stretch of mostly straight and curved roads, while the Urban Area comprises a 1.3 km section densely populated with tall buildings. Each experiment was repeated 10 times per area, and the average RMSE (Root Mean Square Error) values are reported in Table 5.

In the Open Area, there are fewer distinctive features that can be used as landmarks for map matching due to the wide roads and parks. As a result, dynamic object removal based on LiDAR segmentation using DeeplabV3 can introduce errors if objects are incorrectly removed. This is why the RMSE in the Open Area is higher for the LiDAR + NDT map-matching method compared to the conventional approach.

However, when combining vision and LiDAR data to remove dynamic objects, the proposed method showed significant improvements in map-matching performance in both the Open and Urban areas. In the Open Area, the RMSE was improved by approximately 16% compared to the LiDAR-only method, while in the Urban Area, a 15% reduction in RMSE was observed. The proposed method using vision object detection and LiDAR segmentation was able to minimize false positives and missed detections, thus enhancing the overall map-matching accuracy.

## 4. Discussion

- Advantages

The proposed method offers several key strengths:

- Improved localization in complex environments:
  The integration of YOLOv4 with LiDAR enables the effective removal of dynamic obstacles, significantly enhancing the localization accuracy in dense urban areas with heavy traffic.
- Robust object detection and removal:
  YOLOv4’s advanced object detection capabilities reduce common issues such as false positives and missed detections, which often occur with traditional LiDAR segmentation-based methods.

- Disadvantages

While the system demonstrates significant advantages, it also has certain limitations:

- Computational demand for real-time performance:
  The YOLOv4 algorithm, as a learning-based model for dynamic object removal, requires substantial computational power to maintain real-time performance. This may present challenges for implementation in resource-constrained systems, such as standard autonomous vehicles.
- Sensitivity to environmental conditions (parameter sensitivity):
  The system relies heavily on YOLOv4’s camera-based object detection. In environments with poor lighting (e.g., nighttime) or harsh lighting (e.g., strong backlighting from the sun), object detection accuracy decreases, leading to reduced localization performance.
- Trade-off between FPS and accuracy:
  As noted in Table 3, the input size directly impacts the trade-off between FPS and accuracy. Smaller input sizes improve the FPS by reducing the computational load but decrease the accuracy due to the lower resolution. Through experiments, we identified 512 × 512 as the optimal input size, achieving a real-time performance above 10 Hz while maintaining sufficient accuracy for detecting and removing dynamic obstacles. Future work could explore adaptive input sizes to dynamically balance accuracy and speed based on environmental conditions.

## 5. Conclusions

In this study, we presented a novel approach to enhance LiDAR-based mapping and localization for autonomous driving by integrating the YOLOv4 algorithm to effectively remove dynamic objects. Our experimental results demonstrate substantial improvements in both map accuracy and localization performance, particularly in high-traffic urban environments.

We compared three methods: the traditional NDT map-matching approach, a LiDAR segmentation-based method from previous research, and the proposed Vision + LiDAR approach. Across different experimental settings, the proposed method consistently achieved more than a 15% reduction in matching errors compared to previous methods.

Further research is required to address challenges posed by adverse weather conditions, such as rain or fog, which may impact the performance of both the camera and LiDAR sensors. Future work will focus on integrating weather-robust sensor models and developing advanced fusion techniques to enhance the system reliability under such conditions.

## Figures and Tables

**Table 1:** YOLO model comparison (specific to urban dynamic environments).

Model	Number of Parameters	Computational Cost (GFLOPs)	FPS (Tesla V100)	mAP@0.5 (%)	Stability	Environmental Suitability
YOLOv4	64 M	60.9	62	65.7	High	Optimal
YOLOv5-L	46.5 M	109.1	140	66.6	Moderate	Limited
YOLOv7	36.9 M	104.7	161	69.6	Low	Limited

**Table 2:** Performance comparison of object detection algorithms on LiDAR data for vehicle segmentation.

Algorithm	Point net ++	SP Graph	Tangent Conv.	Squeeze SegV2	RangeNet 53++	DeepLab V3+
Size	50,000 pts			64 × 2048 px		
Car	53.7	68.3	86.8	81.8	86.4	92.57
Truck	0.9	0.9	11.6	13.4	25.5	37.3
Other vehicle	0.2	0.8	10.2	14	22.6	67.31

**Table 3:** mAP of YOLOv4 in different input sizes.

Input Size	mAP @ 0.5 (%)	FPS
680 × 608	65.7	21.09
512 × 512	64.9	31.1
416 × 416	62.8	41.0

**Table 4:** Precision of object detection part using YOLOv4 in 512 × 512 size.

Class	Precision (%)	mAP (%)	FPS
Car	83.58	80.42	29.3
Truck	63.81		
Other vehicle	73.27		

**Table 5:** Comparative RMSE values between different methods.

Methods	RMSE		Remarks
	Open Area	Urban Area	
Conventional NDT map matching	0.9870	1.3874	
LiDAR + NDT map matching	1.1623	1.3195	
Vision + LiDAR + NDT map matching	0.9724	1.1217	Proposed method

## Data Availability

Data are contained within the article.
